# Ganglion cell-inner plexiform layer and retinal nerve fiber layer thickness according to myopia and optic disc area: a quantitative and three-dimensional analysis

**DOI:** 10.1186/s12886-017-0419-1

**Published:** 2017-03-11

**Authors:** Sam Seo, Chong Eun Lee, Jae Hoon Jeong, Ki Ho Park, Dong Myung Kim, Jin Wook Jeoung

**Affiliations:** 1 Department of Ophthalmology, Cheil Eye Hospital, Ayang-ro, dong-gu, Daegu, Korea; 2 Department of Ophthalmology, Keimyung University, Dongsan Medical Center, Dongsan-dong, Jung-gu Daegu, Korea; 3 Department of Ophthalmology, Konyang University Hospital, Gasuwon-dong, Seo-gu Daejeon, Korea; 4 Department of Ophthalmology, Seoul National University Hospital, Seoul National University College of Medicine, 101 Daehak-ro, Jongno-gu Seoul, 110-744 Korea

**Keywords:** Ganglion cell inner plexiform layer, Retinal nerve fiber layer, Myopia, Optic disc size, Optical coherence tomography

## Abstract

**Background:**

To determine the influences of myopia and optic disc size on ganglion cell-inner plexiform layer (GCIPL) and peripapillary retinal nerve fiber layer (RNFL) thickness profiles obtained by spectral domain optical coherence tomography (OCT).

**Methods:**

One hundred and sixty-eight eyes of 168 young myopic subjects were recruited and assigned to one of three groups according to their spherical equivalent (SE) values and optic disc area. All underwent Cirrus HD-OCT imaging. The influences of myopia and optic disc size on the GCIPL and RNFL thickness profiles were evaluated by multiple comparisons and linear regression analysis. Three-dimensional surface plots of GCIPL and RNFL thickness corresponding to different combinations of myopia and optic disc size were constructed.

**Results:**

Each of the quadrant RNFL thicknesses and their overall average were significantly thinner in high myopia compared to low myopia, except for the temporal quadrant (all Ps ≤0.003). The average and all-sectors GCIPL were significantly thinner in high myopia than in moderate- and/or low-myopia (all Ps ≤0.002). The average OCT RNFL thickness was correlated significantly with SE (0.81 μm/diopter, *P* < 0.001), axial length (-1.44 μm/mm, *P* < 0.001), and optic disc area (5.35 μm/mm^2^, *P* < 0.001) by linear regression analysis. As for the OCT GCIPL parameters, average GCIPL thickness showed a significant correlation with SE (0.84 μm/diopter, *P* < 0.001) and axial length (-1.65 μm/mm, *P* < 0.001). There was no significant correlation of average GCIPL thickness with optic disc area. Three-dimensional curves showed that larger optic discs were associated with increased average RNFL thickness and that more-myopic eyes were associated with decreased average GCIPL and RNFL thickness.

**Conclusion:**

Myopia can significantly affect GCIPL and RNFL thickness profiles, and optic disc size has a significant influence on RNFL thickness. The current OCT maps employed in the evaluation of glaucoma should be analyzed in consideration of refractive status and optic disc size.

## Background

Glaucoma is a progressive optic neuropathy characterized by loss of retinal ganglion cells (RGCs) and thinning of the retinal nerve fiber layer (RNFL). Therefore, assessment of peripapillary RNFL thickness has been an important approach to the detection of glaucomatous structural damage. Currently, several imaging modalities are available for quantitative analysis of RNFL thickness. One of them, optical coherence tomography (OCT), enables assessment of RGC axons by quantification of RNFL damage. OCT can provide reproducible measurement of RNFL thickness parameters as well as effective differentiation of glaucomatous eyes from healthy eyes [1–3].

Recently, advanced OCT segmentation in the macular region have enabled quantitative evaluation of the individual retinal layers [4–6]. One newly developed OCT algorithm has been shown to have a high level of reproducibility in determining macular ganglion cell-inner plexiform layer (GCIPL) thickness [7]. Considering that more than 50% of the retinal ganglion cells are contained in the macular region, macular assessment offers a theoretical advantage for glaucoma diagnosis. In fact, several studies have demonstrated the usefulness of macular measurement on glaucoma diagnosis [8–11].

Accordingly, OCT has been extensively utilized for diagnosis of, and follow-up on, glaucoma. However, considerable RNFL thickness profile variation among normal healthy subjects has been reported [12, 13]. Recent studies suggest that myopic eyes show thinner RNFL measurements and different RNFL distribution patterns, which can lead to inaccurate diagnostic classification [12, 14–17]. Also, it has been reported that larger optic disc size is associated with thicker RNFL [18, 19]. Although there is substantial evidence of anatomic RNFL variations, it remains to be elucidated whether they have any significant influence on GCIPL thickness measurements. This is a critical issue, because understanding these variations and the causative factors is fundamental to the improvement of OCT-based measurement as a glaucoma-diagnostic test.

The purpose of this study was to determine the influences of myopia and optic disc size on GCIPL and RNFL thickness profiles measured by spectral-domain OCT in young healthy adults.

## Methods

### Subjects

One hundred and sixty-eight healthy subjects, aged between 20 and 32 years, with no other known ocular abnormality and who had undergone health screening, were invited to participate in this study conducted at the Armed Forces Capital Hospital. This study followed the tenets of the Declaration of Helsinki. The Institutional Review Board of the Armed Forces Capital Hospital permitted the review of the subjects’ medical records. The requirement for informed consent by individual patients was waived given the retrospective nature of the study. All data in this study were analyzed anonymously.

All of the subjects underwent a comprehensive ophthalmic examination, including a review of the medical history, measurements of the best corrected visual acuity, manifest refraction, intraocular pressure (IOP) measurement with Goldmann applanation tonometry, gonioscopy, and dilated fundus examination. Refractive error was recorded with an automatic refractometer (RK-F1; Topcon Corporation, Tokyo, Japan). An ocular biometer (Axis II PR; Quantel Medical, Inc., Bozeman, MT, USA) was used to obtain axial length (AL). Photograph of the optic disc and RNFL was obtained with a digital fundus camera (CF-60UVI; Canon, Tokyo, Japan). Confocal scanning laser ophthalmoscopy using the Heidelberg Retina Tomograph II (HRT-II; Heidelberg Engineering, Dossenheim, Germany) was performed to obtain optic disc area. One operator (JHJ) was required to draw a contour line along the disc margin. Heidelberg Eye Explorer (Version 3.1.2), the operating system of the HRT-II, was employed to generate mean topography images and to perform image analysis.

The inclusion criteria were as follows: best-corrected visual acuity of at least 20/40; open anterior chamber angle on gonioscopy; good-quality fundus images; absence of glaucomatous optic neuropathy (GON); absence of RNFL defect according to red-free fundus photography, and reliable normal visual field result. Absence of GON was defined as a cup-to-disc ratio less than 0.6 and an intact neuroretinal rim without optic disc hemorrhages, notches or localized pallor.

The following individuals were excluded: those with a history of ocular trauma or previous ocular surgery, or neurological disease that could have affected the RNFL or GCIPL; those with RNFL defect or disc anomaly; those with any ocular or neurologic disease that could cause visual disturbance; those with a narrow angle on gonioscopic examination; those with clinical features consistent with glaucoma or IOP greater than 21 mmHg in either eye. When both eyes of a candidate subject were eligible for inclusion in the study, one eye was randomly selected.

### Optical coherence tomography

After pharmacologic dilation of the pupil, the subject was seated and properly aligned. Each peripapillary scan centered on the optic disc (optic disc 200 x 200 axial scan) and macular scan centered on the fovea (macular 200 x 200 cube scan) was obtained using a Cirrus OCT device with software version 6.0. The OCT was performed by a single experienced examiner. Scans showing a signal strength greater than 6 and good centration on the optic disc were evaluated.

Utilizing the Fast RNFL program, the RNFL thickness was determined at 256 points around a set diameter (3.4 mm) from the center of the optic disc. The obtained values were averaged to yield 12 clock-hour thicknesses, four-quadrant thicknesses, and a global average RNFL thickness measurement. The clock-hour RNFL thicknesses were recorded based on the right-eye orientation. The superior clock hour was 12 o’clock; the others were assigned accordingly, in the clockwise direction for the right eye and counterclockwise for the left.

The GCA algorithm was employed to detect and measure macular GCIPL thickness within a 14.13 mm^2^ elliptical annulus area centered on the fovea. Average, minimum, and sectoral (superonasal, superior, superotemporal, inferotemporal, inferior, and inferonasal) measurements subsequently were analyzed. Additionally, T/N ratio- the ratio of GCIPL thickness in temporal sectors (sum of superotemporal and inferotemporal) to that in nasal sectors (sum of superotemporal and inferotemporal) - were scrutinized.

### Data analysis

The refraction data were converted to spherical equivalents (SE), and the subjects were assigned to one of three groups according to their SE values: a low-myopia group (-3.0 diopters < SE < 0.0 diopters), a moderate-myopia group (-6.0 diopters < SE ≤ -3.0 diopters), and a high-myopia group (SE ≤ -6.0 diopters). They were also assigned one of three other groups according to their optic disc area values: a small-disc group (disc area ≤ 2.0 mm^2^), a medium-disc group (2.0 mm^2^ < disc area ≤ 2.5 mm^2^), and a large-disc group (disc area > 2.5 mm^2^). The 360° average, quadrant and clock-hour RNFL thickness values and GCIPL thickness values were compared among the groups using multivariate analysis of covariance (MANCOVA). Significant differences in MANCOVA were followed by post-hoc comparisons.

Linear regression analysis was employed to assess the relationship of the SE and/or optic disc area with RNFL or GCIPL thickness values. A statistical analysis was performed with SPSS (version 21.0, SPSS, Chicago, IL, USA), and P values less than 0.05 were considered statistically significant.

Surface plots in the forms of three-dimensional graphs on x-, y-, and z-axes were constructed using SigmaPlot ® Version 11.0 (Systat Software; Inc, Chicago, IL, USA). The graphs were constructed for each measurement, with axes x = disc area (range: 1.3 – 4.2 mm^2^), y = SE (range: -0.5 – –14.0 diopters) and z = RNFL thickness or GCIPL thickness. A local smoothing technique was applied to average the values at neighboring points in order to avoid oscillations and spikes in the surface plots. Each of the resultant surface plots indicated the value of RNFL or GCIPL thickness estimated to yield a different disc area at a different SE. As a visual aid to interpretation, graduated shades of color were used to denote the RNFL or GCIPL thickness values (Fig. 3).

## Results

### Subject characteristics

A total of 168 eyes of 168 subjects were included in the analysis; 92 of these were right eyes. All of the subjects were Korean. The mean subject age was 23.2 ± 2.2 years (range: 20–32 years). The mean SE was -3.58 ± 2.74 diopters (range: -0.50 – -14.00), and the mean axial length was 24.44 ± 1.30 mm (range: 19.88 – 28.07 mm).

### OCT measurements according to degree of myopia

**Fig. 1:**
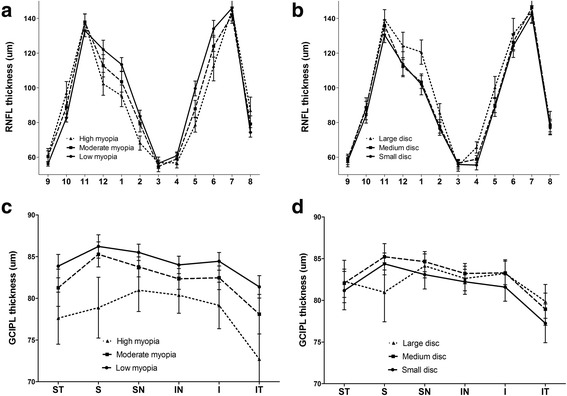
Comparison of Cirrus optical coherence tomography (OCT) clock-hour retinal nerve fiber layer (RNFL) thickness profiles (**a**) among low-, moderate- and high-myopia groups and (**b**) among small-, medium- and large-disc groups. Comparison of Cirrus OCT ganglion cell-inner plexiform layer (GCIPL) thickness profiles (**c**) among low-, moderate- and high-myopia groups and (**d**) among small-, medium- and large-disc groups I, inferior sector; IN, inferonasal sector; IT, inferotemporal sector; S, superior sector; SN, superonasal sector, ST, superotemporal sector

The RNFL and GCIPL thickness measurements of the subgroups, as based on the SE values, are presented in Table 1. A MANCOVA showed that the RNFL and GCIPL parameters were statistically significantly different for the subgroups adjusted for age, IOP, central corneal thickness and disc area (F = 5.487, *p* < 0.001 and F = 2.859, *p* < 0.001, respectively). The average and quadrant RNFL thicknesses were significantly thinner in high myopia than in low myopia, except for the temporal quadrant (all Ps ≤ 0.003). The RNFL thicknesses of the 1, 2, 5, 6, and 12 o’ clock sectors were significantly thinner in moderate and/or high myopia than in low myopia (all Ps < 0.001). By contrast, moderate and/or high myopia showed significantly thicker RNFL values than did low myopia at the 8, 9, and 10 o’clock sectors (*P* = 0.001, 0.003, and < 0.001, respectively) (Fig. 1).

**Table 1:** Retinal nerve fiber layer (RNFL) and ganglion cell-inner plexiform layer (GCIPL) thickness measurements of subgroups based on spherical equivalents (SE)

	Low myopia (LM) (*N* = 86)	Moderate myopia (MM) (*N* = 48)	High myopia (HM) (*N* = 34)	*P* Value	Post hoc
Retinal nerve fiber layer					
Average	96.85 ± 7.49	94.21 ± 7.11	91.59 ± 6.28	**0.003**	LM > HM
Temporal quadrant	70.93 ± 8.07	76.21 ± 14.82	82.32 ± 16.28	**<0.001**	LM < MM/HM
Superior quadrant	122.76 ± 13.57	115.79 ± 19.99	111.82 ± 11.01	**0.001**	LM > MM/HM
Nasal quadrant	67.09 ± 8.49	64.27 ± 9.59	60.53 ± 8.06	**0.001**	LM > HM
Inferior quadrant	126.57 ± 14.02	118.08 ± 11.44	111.74 ± 15.13	**<0.001**	LM > MM/HM
Ganglion cell inner plexiform layer					
Average	84.13 ± 4.39	82.23 ± 4.67	78.24 ± 6.21	**<0.001**	LM/MM > HM
Minimum	81.12 ± 9.75	78.17 ± 12.75	69.12 ± 18.51	**<0.001**	LM/MM > HM
Superotemporal	83.86 ± 6.52	81.27 ± 7.67	77.62 ± 8.94	**0.001**	LM > HM
Superior	86.22 ± 6.50	85.27 ± 5.15	78.88 ± 10.44	**<0.001**	LM/MM > HM
Superonasal	85.51 ± 4.55	83.77 ± 4.12	80.97 ± 7.32	**0.001**	LM > HM
Inferonasal	84.01 ± 4.84	82.33 ± 4.29	80.38 ± 6.23	**0.002**	LM > HM
Inferior	84.44 ± 4.95	82.46 ± 4.87	79.15 ± 7.98	**<0.001**	LM/MM > HM
Inferotemporal	81.38 ± 6.24	78.10 ± 8.46	72.71 ± 8.64	**<0.001**	LM > MM > HM

*P* value by multivariate analysis of covariance (MANCOVA) adjusted by age, intraocular pressure, central corneal thikness, optic disc area

Significant values are shown in bold. Bonferroni’s correction for multiple comparisons

The OCT-measured GCIPL thickness measurements decreased with increasing myopia severity (Table 1, Fig. 1). The average, minimum and all-sectors GCIPL were significantly thinner in highly myopic eyes than in moderate and/or low-myopic eyes (all Ps ≤ 0.002). The temporal/nasal ratio (T/N ratio) showed significantly lower values in the highly myopic eyes (*p* = 0.013), indicating more pronounced GCIPL decreases in the temporal sector.

### OCT measurements according to optic disc area

The RNFL and GCIPL thickness measurements based on the optic disc areas are listed in Table 2. According to a MANCOVA, a statistically significant difference was found in the RNFL parameters between the subgroups adjusted for age, IOP, central corneal thickness, axial length and SE (F = 3.782, *p* < 0.001). Of all OCT RNFL parameters, the superior- and nasal-quadrant RNFL were significantly thicker in the large-disc group than those in the small- and medium-disc groups (*P* ≤ 0.001 and *P* = 0.002, respectively). Also, the clock-hour thicknesses were significantly greater in the large-disc group, specifically in the 12, 1, 2, 4, and 5 o’ clock sectors, than in the small- and/or medium-disc group (all Ps < 0.05). By contrast, none of the OCT GCIPL parameters significantly differed among the three optic disc groups.

**Table 2:** RNFL and GCIPL thickness measurements based on optic disc areas

	Small Disc (S) (*N* = 46)	Medium Disc (M) (*N* = 83)	Large Disc (L) (*N* = 39)	*P* Value	Post hoc
Retinal nerve fiber layer					
Average	92.41 ± 5.83	94.48 ± 6.81	99.28 ± 8.57	**<0.001**	S/M < L
Temporal quadrant	73.37 ± 11.02	75.21 ± 12.44	75.36 ± 15.93	0.760	
Superior quadrant	115.30 ± 12.75	115.84 ± 16.34	128.15 ± 14.46	**<0.001**	S/M < L
Nasal quadrant	62.52 ± 7.04	64.53 ± 9.62	68.74 ± 8.88	**0.002**	S/M < L
Inferior quadrant	117.98 ± 13.50	121.04 ± 13.64	125.10 ± 17.68	0.054	
Ganglion cell inner plexiform layer					
Average	81.52 ± 4.96	82.83 ± 5.58	82.49 ± 5.32	0.426	
Minimum	77.33 ± 10.47	78.56 ± 14.11	76.95 ± 15.54	0.809	
Superotemporal	81.17 ± 7.74	82.05 ± 7.77	82.26 ± 7.81	0.772	
Superior	84.37 ± 4.41	85.22 ± 7.21	82.97 ± 10.91	0.426	
Superonasal	83.09 ± 5.83	84.64 ± 5.50	84.13 ± 4.44	0.374	
Inferonasal	82.22 ± 5.02	83.21 ± 5.55	82.62 ± 4.52	0.654	
Inferior	81.59 ± 5.70	83.29 ± 6.48	83.21 ± 5.10	0.221	
Inferotemporal	77.24 ± 7.82	78.94 ± 8.82	79.87 ± 6.25	0.304	

*P* value by multivariate analysis of covariance (MANCOVA) adjusted by age, intraocular pressure, central corneal thickness, myopia, axial length

Significant values are shown in bold. Bonferroni’s correction for multiple comparisons

### Effects of myopia and optic disc area on OCT measurements

**Fig. 2:**
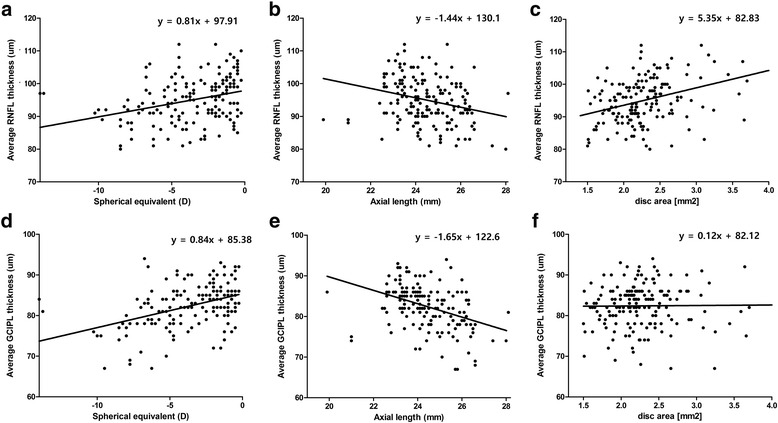
Scatterplot of simple linear regression between average RNFL thickness and (**a**) spherical equivalent (SE) (*p* < 0.001), (**b**) axial length (*p* < 0.001), (**c**) optic disc area (*p* < 0.001). Scatterplot showing average GCIPL thickness against (**d**) SE (*p* < 0.001), (**e**) axial length (*p* < 0.001), (**f**) optic disc area (*p* = 0.895)

The simple linear regression analysis results revealed average OCT RNFL thickness to be correlated significantly with SE (0.81 μm/diopter, *P* < 0.001, Fig. 2a), axial length (-1.44 μm/mm, *P* < 0.001, Fig. 2b), and optic disc area (5.35 μm/mm^2^, *P* < 0.001, Fig. 2c). Additionally, the linear regression analysis of the OCT GCIPL parameters showed average GCIPL thickness to be in significant correlation with SE (0.84 μm/diopter, *P* < 0.001, Fig. 2d) and axial length (-1.65 μm/mm, *P* < 0.001, Fig. 2e). There was, however, no significant correlation between average GCIPL thickness and optic disc area (Fig. 2f).

Associations among GCIPL, RNFL thickness, myopia and disc area were evaluated by multiple linear regression, with OCT parameters as the dependent variable and age, IOP, axial length, SE, disc area, and central corneal thickness as the independent variables. The following equations represent the effects of myopia and disc area on GCIPL and RNFL thicknesses.

$$ RNFL\  thickness\ \left(\mu m\right) = 97. 805 + 5. 487 \times disc\  area\ \left( m{m}^2\right) + 0. 896 \times spherical\  equivalent\ (diopter) - 0. 499 \times age\ (year) $$

$$ GCIPL\kern0.5em  thickness\left(\mu m\right)= 85. 379+ 0. 835 \times spherical\kern0.5em  equivalent\kern0.5em (diopter) $$

From the equation, only SE demonstrated significant effects on average GCIPL thickness. Age and disc area did not show significant effects on average GCIPL thickness, but were associated with average RNFL thickness. IOP, axial length and central corneal thickness had no significant effects on RNFL or GCIPL thickness, based on the multiple regression analysis.

**Fig. 3:**
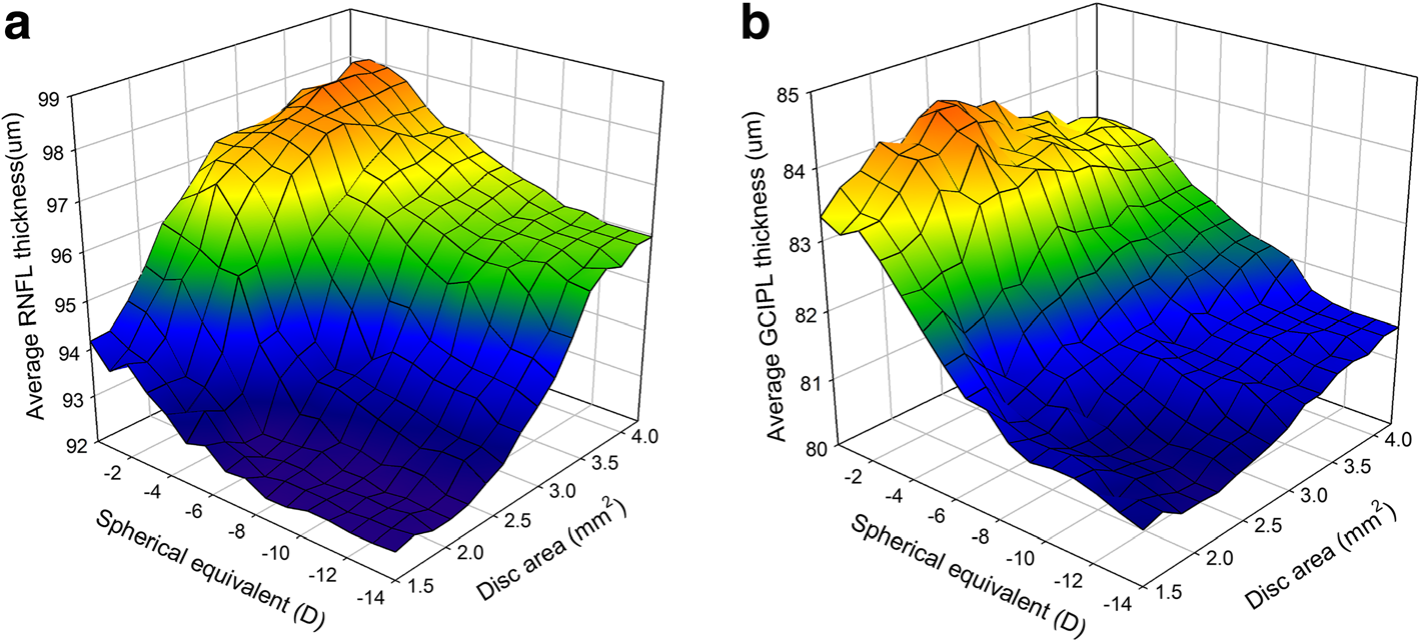
**a** Relationship of SE and/or optic disc area with average RNFL thickness. The RNFL thickness graph shows an RNFL decrease associated with a combination of high myopia and small optic disc area. Also, the effect of the optic disc area on RNFL is more pronounced than that of the SE. **b** Relationship of SE and/or optic disc area with average GCIPL thickness. The GCIPL thickness graph slopes from the highest point in low myopia to the lowest point in high myopia. This reflects the correlation of GCIPL thickness decrease with myopic degree

Three-dimensional graphs of the OCT RNFL and GCIPL thickness values show the combined influence of myopia and disc size (Fig. 3). Larger optic discs were associated with increasing average RNFL and GCIPL thickness, and the effect of optic disc area was more pronounced in the OCT RNFL parameters than in the OCT GCIPL ones. Also, more-myopic eyes were associated with decreasing average RNFL and GCIPL thickness, and the effect was less pronounced in the OCT RNFL parameters than in the OCT GCIPL ones.

## Discussion

Previous studies have reported significant relationships between RNFL thickness and several factors such as age, gender and axial length [20–23]. In the present study, we confirmed an association between decreasing OCT RNFL thickness and more-myopic eyes, which finding accords well with some previous studies [14, 15, 24]. Additionally, this study found that RNFL thickness decreased with increasing myopia degree, except for the temporal sector, and that the high-myopia subgroups had thicker temporal RNFL. These results indicate that RNFL redistribution might occur as the axial length increases, which mechanism has been posited in several previous papers [15, 25, 26]. Kim et al. speculated that with increasing axial length, the retina is dragged toward the temporal horizon, which might result in RNFL thickening in the temporal quadrant in myopic eyes [16].

The literature has offered relatively little information regarding the effect of myopia on GCIPL thickness profiles. Choi et al. reported significantly thinner average GCIPL thicknesses in highly myopic eyes, though some areas showed no association with refraction [27]. Koh et al. reported a correlation of macular GCIPL thickness with axial length [28]. Our present results showed thinner GCIPL thicknesses in myopic eyes, which effect was evenly distributed throughout the sectors. We also found a negative correlation between average GCIPL thickness and axial length.

As for GCIPL thickness, decreased T/N ratio in myopic eyes has implied more distinct GCIPL thinning in the temporal part of the macula. Decreasing GCIPL thickness with increasing myopia can be explained by the stretching effect from an elongated eye. That is, as the globe elongates in myopic eyes, the larger retinal surface area results in a lower ganglion cell density. This effect would be more significant toward the periphery. It is conceivable that the nasal part of the macula, which is relatively near to the center of the disc, might be less affected.

It is noteworthy that the effect of optic disc area, in the present study, was more pronounced in the RNFL parameters than in the GCIPL ones. Although this issue has not been fully elucidated, some explanations can be posited. Greater RNFL thickness in large-disc eyes might be attributable to overestimation related to the scanning circle distance. To elaborate, as RNFL thickness decreases with increasing distance from the disc margin, a shorter distance between the scanning circle and the disc margin in large discs would result in RNFL thickness overestimation [16]. Also, it is possible that, as established in previous histomorphometric studies, eyes with a larger optic disc area retain more retinal nerve fiber axons [29, 30]. However, GCIPL measurement yields a relatively constant value irrespective of optic disc size variability. Furthermore, macular GCIPL measurement includes only a portion of the RGC axons in the central macular area, whereas peripapillary RNFL analysis accounts for all of them [7, 31]. A recent study demonstrated only fair correlations between macular GCIPL and optic disc or RNFL parameters by spectral domain OCT [32]. This regional difference might lead to GCIPL and RNFL result discrepancies.

The main goal of this study was to evaluate the influence of myopia, optic disc size and their combined effect on OCT parameters. It has some notable strengths compared with previous studies. First, only young healthy subjects (mean age: 23.2 ± 2.2 years; range: 20–32 years) were enrolled. Thereby, the aging effect on OCT parameters could be minimized. That RNFL and GCIPL thicknesses become systematically thinner with age has been well established [7, 33–35]. This is to say that the association between myopia and/or optic disc area and OCT measurement might differ according to the subject’s age. In this light, we believe that the current study, by minimizing the aging effect, provides uncommonly clear insights into the effects of myopia and optic disc size on OCT parameters. The second significant strength of this study is its provision of three-dimensional graphs that represent the combined influence of myopia and optic disc size on OCT parameters. Our results, therefore, indicate the importance of careful interpretation of the current OCT maps in cases of eyes of varying myopic degree and disc size.

This study has some limitations. First, ocular magnification can partially affect RNFL and GCIPL thickness measurements. However, it should be noted that several studies have shown conflicting results regarding the relationship between myopia and OCT RNFL/GCIPL parameters after magnification correction [36–38]. Additional studies investigating this relationship might be needed. Second, we included only young healthy subjects of uniform age and ethnicity in order to eliminate potential confounding factors. Further investigation should focus on subjects of other age groups and/or ethnicities.

In conclusion, myopia and optic disc size can significantly affect OCT RNFL and GCIPL thickness profiles. Larger optic discs are associated with increased RNFL thickness, and more-myopic eyes are associated with decreasing average RNFL and GCIPL thicknesses. Clinicians should recognize that the current OCT maps employed in the evaluation of glaucoma should be analyzed considering refractive status and optic disc size.

## Conclusions

This study investigated the effect of myopia and optic disc size on the ganglion cell inner plexiform layer (GCIPL) and retinal nerve fiber layer (RNFL) thickness profiles. RNFL and GCIPL thickness profiles were affected by the refractive error and optic disc size. RNFL and GCIPL analysis in the evaluation of glaucoma should always be interpreted with reference to the refractive status and optic disc size.

## Abbreviations

GCIPL: Ganglion cell-inner plexiform layer
GON: Glaucomatous optic neuropathy
IOP: Intraocular pressure
OCT: Optical coherence tomography
RGC: Retinal ganglion cell
RNFL: Retinal nerve fiber layer
SE: Spherical equivalent
SE: Spherical equivalents
T/N ratio: The ratio of GCIPL thickness in temporal sectors to that in nasal sectors
